# Correction: Elucidation of hydrogen bonding formation by a computational, FT-IR spectroscopic and theoretical study between benzyl alcohol and isomeric cresols

**DOI:** 10.1039/d2ra90121h

**Published:** 2022-12-01

**Authors:** M. Raveendra, M. Chandrasekhar, C. Narasimharao, L. Venkatramana, K. Siva Kumar, K. Dayananda Reddy

**Affiliations:** Department of Chemistry, P.V.K.N. Govt. Degree & P.G. College Chittoor 517001 A.P. India; Department of Physics, Vignan Institute of Technology and Science Deshmukhi Telagana India; Department of Chemistry, S.V. University Tirupati 517502 A.P. India; Department of Chemistry, Indian Institute of Technology Madras Chennai 600 036 India; Department of Chemistry, S.V. Arts Degree & P.G. College (T.T.D'S) Tirupati 517502 A.P. India sivakumarkasi64@gmail.com +91-9290080843

## Abstract

Correction for ‘Elucidation of hydrogen bonding formation by a computational, FT-IR spectroscopic and theoretical study between benzyl alcohol and isomeric cresols’ by M. Raveendra *et al.*, *RSC Adv.*, 2016, **6**, 27335–27348, https://doi.org/10.1039/C5RA26298D.

The authors regret that the name of one of the authors (L. Venkatramana) was shown incorrectly in the original article. The corrected author list is as shown above.

The Royal Society of Chemistry apologises for these errors and any consequent inconvenience to authors and readers.

## Supplementary Material

